# HIV-1 infection among women in Israel, 2010–2018

**DOI:** 10.1186/s12879-020-05389-6

**Published:** 2020-09-07

**Authors:** Tali Wagner, Karen Olshtain-Pops, Marina Wax, Olivia Horwitz, Rachel Shirazi, Yael Gozlan, Hadar Volnowitz, Ella Mendelson, Itzchak Levy, Orna Mor

**Affiliations:** 1 Sackler Faculty of Medicine, Tel-Aviv University, Tel-Aviv-Yafo, Israel; 2 National HIV and Viral Hepatitis Reference Laboratory, Chaim Sheba Medical Center, Tel Hashomer, 52621 Ramat Gan, Israel; 3 Hadassah Medical Center, Jerusalem, Israel; 4 Infectious Disease Unit, Chaim Sheba Medical Center, Ramat Gan, Israel

**Keywords:** HIV-1, Women, Resistance-testing, Transmitted drug resistance mutations (TDRM)

## Abstract

**Introduction:**

Although women comprise 33% of the HIV-1-carriers in Israel, they have not previously been considered a risk group requiring special attention. Immigration waves from countries in Africa and in East Europe may have changed the local landscape of women diagnosed with HIV-1. Here, we aimed to assess viral and demographic characteristics of HIV-1-positive women identified in Israel between 2010 and 2018.

**Methods:**

All > 16 year-old, HIV-1-infected women, diagnosed in Israel in 2010–2018, (*n* = 763) registered in the National HIV reference laboratory were included in this cross-sectional study. Demographic and clinical characteristics were extracted from the database. Viral subtypes and transmitted drug resistance mutations (TDRM) were determined in 337 (44.2%) randomly selected samples collected from treatment-naive women.

**Results:**

Median age at diagnosis was 38 years. Most (73.3%) women were immigrants from the former Soviet Union (FSU) (41.2%, 314) or sub-Saharan Africa (SSA) (32.2%, 246) and carried subtype A (79.7%) or C (90.3%), respectively. Only 11.4% (87) were Israeli-born women. Over the years, the prevalence of women from SSA decreased while that of women from FSU increased significantly (*p* < 0.001). The median CD4^+^ cell count was 263 cells/mm^3^, and higher (391 cells/mm^3^) in Israeli-born women. TDRM were identified in 10.4% of the tested samples; 1.8, 3 and 7.1% had protease inhibitors (PI), nucleotide reverse transcriptase inhibitors (NRTI) and non-nucleoside reverse transcriptase inhibitors (NNRTI) TDRM, respectively. The prevalence of women with NNRTI TDRM significantly increased from 4.9% in 2010–2012 to 13.3% in 2016–2018. Israeli-born women had the highest prevalence (16.3%) of NNRTI TDRM (*p* = 0.014). NRTI A62 (5.6%), NNRTI E138 and K103 (5.6 and 4.2%, respectively) were the most prominent mutated sites.

**Conclusions:**

Most HIV-1-positive women diagnosed in Israel in 2010–2018 were immigrants, with the relative ratio of FSU immigrants increasing in recent years. The high proportion of women diagnosed with resistance mutations, particularly, the yearly increase in the frequency of NNRTI mutations, support the national policy of resistance testing at baseline.

## Introduction

Women comprise more than half (51.2%) of the 36.7 million people worldwide carrying HIV-1 [1]. However, the proportion of newly infected women varies around the world [2], with the majority (56%) living in Sub–Saharan Africa (SSA) [2], a region suffering from a generalized HIV-1 epidemic (> 1% HIV-1 prevalence) [3]. The second major region with a high proportion of HIV-1-positive women (42%) is Eastern Europe, particularly countries in the Former Soviet Union (FSU), which experienced the fastest growing HIV-1 epidemic in the world [4] between 2003 and 2009, and is currently regarded as a region of concentrated HIV-1 infection [3].

Interventions aiming to reduce the global spread of HIV-1 require understanding modes of HIV-1 transmission, viral subtype distribution and circulation of drug-resistant viruses. Viruses harboring drug resistance mutations are a major obstacle to successful HIV treatment, even in the current era of HIV treatment simplification and the shift to dual therapy regimens [5, 6]. Immigrants from countries with high rates of HIV-1 infection and of viruses with resistance mutations, may be infected and continuously transmit drug-resistant viruses after immigration [7].

Israel is a multicultural country with a continuous influx of immigrants from across the globe. Until 2010, as a result of massive immigration waves, 41.3% of all HIV cases were immigrants from SSA [8]. Between 2010 and 2018, 174,934 people immigrated to Israel, more than of 50% of whom were women [9, 10]. During this period, most immigrants (59.5%, 104,086) [9, 10] were from the FSU. In comparison, immigrants from SSA [9] constituted only 6.7% of the total number of immigrants in 2010–2017 (9829/146,835), with a decline from 1918 immigrants in 2010 to 318 in 2017.

Gender is known to be a factor that significantly impacts migration experiences [11]. As a result of economic insecurity, limited education, linguistic and cultural barriers, migrants most often present late to care. These factors may also place migrants, especially women, at risk for acquiring HIV-1 infection [12]. Women immigrating from countries with high rates of HIV-1 infection, unaware of their HIV status, are also at higher risk for delivering infants with perinatally acquired HIV-1 [13], especially as in Israel, where universal HIV-1 prenatal screening is not mandatory [14].

According to the Israeli Ministry of Health, women comprise 33% of the reported HIV-1-positive individuals [15]. In a report that summarized HIV-1 diagnosis in Israel between 1981 and 2010, most HIV-1-positive women were from countries in Africa, mainly from Ethiopia. Those infected by injecting drugs or through heterosexual transmission comprised only a small minority of the reported cohort [8]. The characteristics of HIV-1 positive women population and the rate of transmitted resistance mutations (TDRM) in women diagnosed in more recent years have not been evaluated. The goal of this study was to profile the demographic and viral characteristics of HIV-1-positive women diagnosed between 2010 and 2018, and to estimate the proportion of women carrying HIV-1 TDRM in Israel.

## Methods

In this cross-sectional study, the database of the National HIV Reference Center, which has demographic and clinical documentation on all newly diagnosed HIV-1 patients in Israel, was screened for women diagnosed between January 2010 and December 2018. Men, trans-people, women below the age of 16 years and women diagnosed in years other than 2010–2018 were excluded. Demographic (age, birth place and route of HIV-1 transmission) and clinical (year of HIV diagnosis, HIV-1 viral load, CD4^+^ cell counts, HIV-1 subtype and TDRM) characteristics were collected.

The final cohort included 763 women. As not all treatment-naïve, HIV-1-positive women are routinely tested for resistance, the first available sample collected < 6 months after initial HIV-1 diagnosis of 337 women (44.2%), selected each year by a stratified random selection design were analyzed by sequencing of HIV-1 protease (PR, codons 4–99) and reverse transcriptase (RT, codons 38–247). PR and RT TDRM were determined using the World Health Organization (WHO) consensus list of drug resistance mutations updated in 2009 [16] in the HIVdb Program v.8.8 [17]. The polymorphic RT-E138 and accessory mutation A62 sites were also assessed. Subtypes were defined by the REGA HIV-1 subtyping tool version 3.0 and Stanford University HIV Drug-Resistance Database [17].

Descriptive statistics was used to assess the study cohort. Variables with non-Gaussian abnormal distribution (assessed by Kolmogorov-Smirnov test) were expressed by median and interquartile range and the Kruskal Wallis test was performed to test the quality of means of several distributions. Categorical variables were expressed by frequencies and compared using chi–squared or Fisher’s exact test. The Bonferroni method was applied to check whether multiple testing could lead to the risk of type 1 errors. Logistic regression was used to test factors associated with TDRM rates. Multivariable analysis included factors found to be related (*p* < 0.01) to the dependent variable with the forward technique covariate selection and was based on unstandardized effect-size statistics. Potential interactions were controlled by stratification on effect-measure-modifiers to assess heterogeneity of a measure across the levels of another factor. Variables with missing values (e., g missing CD4 results) were ignored. Poisson segmented regression (that typically aggregates individual-level data by time points and estimates dynamic changes over time, while adjusting for secular changes [18]) was performed to examine the change in the frequency of HIV TDRM in the study years. Statistical analysis was performed using IBM SPSS statistics version 20.

## Results

Table 1 summarizes the baseline characteristics of HIV-1-positive women diagnosed in Israel in the years 2010–2012, 2013–2015 and 2016–2018. Median age at diagnosis was 38. Main route of HIV transmission (82.4%, *n* = 629) was sexual contact; only 10.9% (*n* = 83) were injecting drug users (IDUs). Most women were immigrants: 41.2% (*n* = 314) were born in the FSU, 32.2% (*n* = 246) in SSA and only 11.4% (*n* = 87) were born in Israel. While the total number of women identified remained stable over the study period, a significant yearly decline in the proportion of SSA immigrants versus a constant increase in women originating from the FSU was observed (*p* < 0.001). Similarly, while the overall prevalence of subtype C (41.8%, 141/337) and A (38.6%, 130/337) diagnosis was similar, the later years of the study were associated with a decline in the number of subtype C carriers and an increase in the number of subtype A carriers.

**Table 1:** Characteristics of women diagnosed with HIV, Israel, 2010–2018

	All years (2010–2018) *N* = 763	2010–2012 *N* = 246	2013–2015 *N* = 257	2016–2018 *N* = 260	*p value*
Median age at diagnosis (IQR)	38 (31–46)	37 (29–43)	37 (30–46)	40 (34–48)	< 0.001
Place of birth, n (%)					
SSA	246 (32.2)	110 (44.7)	64 (24.9)	72 (27.7)	
FSU	314 (41.2)	77 (31.3)	107 (41.6)	130 (50)	
Israel	87 (11.4)	30 (12.2)	32 (12.5)	25 (9.6)	< 0.001
Other/Unknown	116 (15.2)	29 (11.8)	54 (21)	33 (12.7)	
Risk Groups, n (%)					
Sexual contact	629 (82.4)	202 (82.1)	211 (82.1)	216 (83.1)	
IDU	83 (10.9)	34 (13.8)	33 (12.8)	16 (6.2)	0.001
Other/Unknown	51 (6.7)	10 (4.1)	13 (5.1)	28 (10.8)	
HIV-1 Subtype (N)	337	123	109	105	
A, n (%)	130 (38.6)	45 (36.6)	40 (36.7)	45 (42.9)	
B, n (%)	34 (10.1)	10 (8.1)	17 (15.6)	7 (6.7)	
C, n (%)	141 (41.8)	61 (49.6)	44 (40.4)	36 (34.3)	0.014
G/AG, n (%)	23 (6.8)	3 (2.4)	7 (6.4)	13 (12.4)	
Other, n (%)	9 (2.7)	4 (3.3)	1 (0.9)	4 (3.8)	
CD4 (cells/mm*3) (*n* = 171), median (IQR)	263 (121–466)	285 (146–496)	270 (133–492)	234 (73–394)	0.283
VL (Log c/mL) (*n* = 236), median (IQR)	4.5 (3.9–5.4)	4.4 (3.7–5.4)	4.6 (4.1–5.2)	4.8 (4.3–5.3)	0.519

Data are presented as n (%) or median (IQR); *IQR* Interquartile range, *VL* Viral load, *SSA* Sub-Saharan Africa, *FSU* Former Soviet Union, *IDU* Injecting drug users.

A comparison of the characteristics of women born in SSA, FSU, Israel or elsewhere (Table 2) showed that most women from the FSU (79.7%) were carriers of subtype A, while 90.3% of those from SSA carried subtype C (*p* < 0.001). Women were diagnosed with low (< 350 cells/mm*3) CD4^+^ cell counts (Table 1), with lower median counts among women immigrating from SSA and the FSU (246 cells/mm*3 and 262 cells/mm*3, respectively) as compared to Israeli-born women (391 cells/mm*3, *p* = 0.042, Table 2).

**Table 2:** Characteristics of women diagnosed with HIV in 2010–2018, by place of birth

	SSA	FSU	Israel	Other	*p* value
HIV-1 Subtype (*N* = 337)	*n* = 124	*n* = 123	*n* = 43	*n* = 47	
A, n (%)	2 (1.6)	98 (79.7)	14 (32.6)	16 (34)	< 0.001
C, n (%)	112 (90.3)	8 (6.5)	4 (9.3)	17 (36.2)	
Non A, C, n (%)	10 (8.1)	17 (13.8)	25 (58.1)	14 (29.8)	
CD4 (cells/ mm*3, *N* = 159)	*n* = 52	*n* = 81	*n* = 26	No data	
Median (IQR)	246 (99–348)	262 (106–488)	391 (179–738)		0.042
TDRM by class (*N* = 337)					
All TDRM, n (%)	16 (12.9)	10 (8.1)	7 (16.3)	2 (4.3)	0.170
NNRTI, n (%)	11 (8.9)	6 (4.9)	7 (16.3)	0	0.014
NRTI n (%)	5 (4)	4 (3.3)	0	1 (2.1)	0.582

Data are presented as n (%) or median (IQR-interquartile range); Significance for differences was measured using chi-squared test, Fisher’s Exact test, or Kruskal-Wallis test. TDRMs-transmitted drug resistance mutations; NNRTI-non nucleoside reverse transcriptase resistance mutations; NRTI-nucleotide reverse transcriptase resistance mutations.

**Fig. 1:**
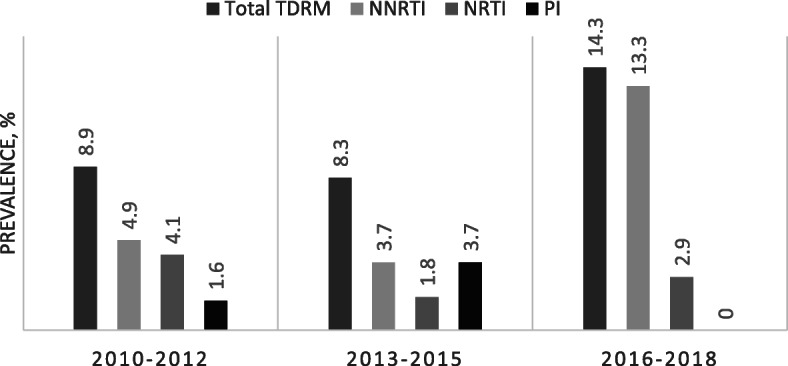
Prevalence of women diagnosed with HIV-1 TDRM in 2010–2012, 2013–2015 and 2016–2018. Prevalence of women with any TDRM (total) and with NRTI, NNRTI and PI TDRM

Resistance analysis revealed that 10.4% (35/377) of women carried viruses with resistance mutations, with 7.1, 3, and 1.8% of women carrying NNRTI, NRTI and PI TDRM, respectively. While the proportion of women with NNRTI TDRM increased significantly (*p* = 0.017) between 2010 and 2012 and 2016–2018, paralleling a non-statistically significant increase in the overall prevalence of women with any HIV-TDRM diagnosed in these years, the rates of women with NRTI and PI TDRM remained stable. Moreover, in 2016–2018, no women with PI TDRM were identified (Fig. 1). All these results were further corroborated by Poisson segmented regression. No significant difference was observed in the prevalence of women with any TDRM between the different birth-places (*p* = 0.170). Interestingly, the proportion of native Israeli women born in carrying a NNRTI TDRM virus (16.3%, *p* = 0.014) was significantly higher compared its prevalence among women born in other countries (Table 2).

Logistic regression was used to assess factors associated with TDRM carriage and carriage of specific TDRMs by drug class. Factors included in this analysis were birthplace (FSU, SSA, Israel or other), HIV-1 subtype (A, C or non A/C), viral load, age at diagnosis and year of diagnosis (supplemental Table S1). Significant association between recent diagnosis and NNRTI TDRM as found by both univariate (OR: 1.23, 1.05–1.45 of 95% CI, *p* = 0.01) and multivariate analysis (OR: 1.23, 1.03–1.43 of 95% CI, *p* = 0.020). Other associations could not be found.

Table 3 lists the type of TDRMs identified in the study cohort according to drug class. The polymorphic RT-E138 and the accessory mutation A62 sites, were also included due to their clinical relevance and high prevalence. A62V which was the most prominent NRTI mutation (5.6%, 19/337), was significantly more common in HIV-1 subtype A- as compared to HIV-1 subtype C-infected women (13%, 17/130 versus 1.4%, 2/141, *p* < 0.001). E138 was the most frequently identified mutated NNRTI position (5.6%, 19/337), detected in 8.5% (*n* = 11), 4.3% (*n* = 6), 3% (*n* = 1) and 4.3% (*n* = 1) of subtype A, C, B and G/AG carriers, respectively. The NNRTI K103N/S mutation was identified in 4.2% (14/337) of women, and was significantly more prominent in those carrying HIV-1 subtype B compared to those carrying subtype C (11.8%, 4/34 versus 2.1%, 3/141, *p* = 0.010). The most prominent PI mutation was M46I, identified in 1.5% (5/337) of patients.

**Table 3:** Prevalence of most frequently detected TDRM (including NRTI A62 and NNRTI E138) in women, 2010–2018

Drug Class	DRM	HIV-1 Subtype						***p value***	***p value***	***p value***
		All	A	C	B	G/AG	Other	***A*** vs. ***C***	***B*** vs. ***C***	***A*** vs. ***B***
		***N*** = 337	***N*** = 130	***N*** = 141	***N*** = 34	***N*** = 23	***N*** = 9			
PI, n (%)	D30N	1 (0.3)		1 (0.7)						
	M46I	5 (1.5)	1 (0.8)	4 (2.8)				*0.222*		
	V82MS	1 (0.3)				1 (1.5)				
NRTI, n (%)	M41L	1 (0.3)		1 (0.7)						
	A62V	19 (5.6)	17 (13)	2 (1.4)				*< 0.001*		
	D67EGN	5 (1.5)	3 (2.3)	2 (1.4)				*0.582*		
	K70R	3 (0.9)	2 (1.5)	1 (0.7)				*0.526*		
	M184V	5 (1.5)	3 (2.3)	2 (1.4)				*0.582*		
	T215EIS	4 (1.2)	1 (0.8)	3 (2.1)				*0.376*		
	K219Q	1 (0.3)	1 (0.8)							
NNRTI, n (%)	K101E	1 (0.3)		1 (0.7)						
	K103NS	14 (4.2)	5 (3.8)	3 (2.1)	4 (11.8)	2 (8.7)		*0.407*	*0.010*	*0.068*
	V106M	1 (0.3)		1 (0.7)						
	E138AGKQ	19 (5.6)	11 (8.5)	6 (4.3)	1 (3)	1 (4.3)		*0.156*	*0.731*	*0.276*
	Y181C	5 (1.5)	1 (0.8)	4 (2.8)				*0.222*		
	Y188L	1 (0.3)	1 (0.8)							
	G190AS	6 (1.8)	2 (1.5)	4 (2.8)				*0.465*		

Data are presented as n (%). Differences in proportions were measured using the chi-squared test. Empty cells, n = zero.

## Discussion

Analysis of the demographic profiles of women diagnosed with HIV-1 in Israel between the years 2010 and 2018 revealed that most were not born in Israel. In 2010–2012, 44.7% were immigrants from SSA and 31.3% were from the FSU. In more recent years (2016–2018), 50% were from the FSU, while only 27.7% originated from SSA (*p* < 0.001). The most prevalent viral subtype, changed accordingly, from subtype C, characteristic of HIV-1 in SSA, in 2010–2012, to subtype A, characteristic to FSU, in 2016–2018 (*p* < 0.014). These results are in concordance with the waves of immigration from SSA and Eastern Europe to Israel in 2010–2018. A similar increase in the prevalence of subtype A carriers was recently reported in Germany and in other west-European countries, due to an increased flow of refugees, mainly from the FSU, into Europe, and especially into Germany [19].

The low CD4 counts noted in this cohort of HIV-positive women, suggest late diagnosis. Moreover, women from SSA, as well as those who immigrated from the FSU, had significantly lower CD4 counts at diagnosis compared to Israeli-born women. Missed opportunities for early diagnosis has already been reported for at least 33% of the Israeli HIV population [20]. Late diagnosis was also recently reported to characterize over half of the women diagnosed in Europe in 2018 [21]. Our data corroborate these results and highlight the need for improved HIV diagnosis policies targeting new female immigrants. These can include offering HIV testing soon after the arrival of all women immigrating from concentrated and generalized HIV epidemic regions, such as the FSU and SSA, respectively. Also, as most of the women are diagnosed at the reproductive age (median age at diagnosis was 38 years), universal testing for HIV-1 infection during pregnancy should be employed, without limiting it to a selected group, e.g., immigrants from SSA, as is currently performed [22]. It was already demonstrated that a universal approach to perinatal HIV testing achieves the best health outcomes and is cost-effective across a range of HIV-1 prevalence settings [23].

TDRMs were identified in 10.4% of women diagnosed in the years 2010–2018. Prevalence of women with NNRTI, NRTI and PI TDRMs was 7.1, 3 and 1.8%, respectively. The proportion of women diagnosed with any TDRM and especially with NNRTI TDRMs increased significantly in more recent years, reaching 14.4 and 13.3%, respectively, among women diagnosed in 2016–2018. In a recent analysis of HIV diagnoses in 2017 in 9 European countries, the overall prevalence of resistance mutations in treatment-naïve patients was 13.5% and that of NNRTI was 7.7% [24]. Although these results are similar to our findings in women, they are likely an overestimation of the actual TDRM rate in Europe, as all resistance mutations included in the Stanford HIVdb were considered [16, 17]. In general, changes in prescribing practices over the study period, the high genetic barrier of PI and the lower genetic barrier of NNRTIs, most likely explain the changing rates of drug class-related TDRMs [25]. However, the overall high rate of resistance mutations, the ongoing increase in transmission of resistant viruses, especially in more recent years, and the high rate of individuals on antiviral therapy worldwide, mandates continuous monitoring of pretreatment resistance mutations in Israel and around the world.

NNRTIs are not considered preferred first-line therapies, but are still included in at least some regimens [5, 25]. In the current study, K103N/S, which confers high-level or intermediate cross-resistance to the NNRTIs efavirenz, nevirapine and delavirdine, was the most prominent NNRTI TDRM (4.2%) and more prevalent in HIV-1 subtype B carriers, as previously reported [26]. As current guidelines permit the use of efavirenz among women of childbearing potential, this rather frequent TDRM should not be disregarded. The polymorphic E138 was the most frequently mutated NNRTI site. This naturally occurring polymorphism that blocks the NNRTI-binding pocket, is known to affect rilpivirine binding and may cause lower susceptibility to this drug [27]. A systematic review that assessed the prevalence of rilpivirine-related TDRMs in 65 countries, already reported an association between E138 mutations and HIV-1 subtypes C (6.1%), and A (3.3%) [28]. In the current study, it was identified in 5.6% of all women, irrespective of the viral subtype. As rilpivirine-based dual therapy is still considered a legitimate treatment option, resistance testing in all patients prior to rilpivirine therapy should be performed. The most prominent NRTI accessory non-polymorphic mutated site was A62V (5.6% prevalence), which influences replication fidelity and viral fitness in the context of multi-drug resistance mutations [17]. A62V, which was reported to be widespread in subtype A viruses in the FSU [17], was also significantly more prominent in HIV-1 subtype A in the present analysis. However, according to current guidelines, A62V does not interfere with therapy.

While there was no significant difference between overall TDRM rates in women originating from different countries, significantly higher NNRTI TDRM rates (16.3%) were identified in women born in Israel compared to those born in SSA (8.9%) or FSU (4.9%, *p* = 0.014). In an earlier study that assessed HIV-positive patients diagnosed between 1999 and 2003 in Israel, resistance mutations were reported in 14.8% of newly diagnosed, treatment-naïve patients, 28.6% of whom were known to have been infected in Israel [7]. Together, these results suggest continuous ongoing local circulation of drug-resistant viruses. An in-depth characterization of all HIV-1 patients identified in 2010–2018 is ongoing.

Our study has several limitations. The main inherent limitation was the overall small number of women positive for HIV diagnosed in Israel. Also, resistance analysis was not performed for all women. However, a stratified selection design was used to selected samples from each year for sequencing and TDRM analysis. However, this study was the first to focus on women diagnosed with HIV in Israel. Women are a subgroup of patients not previously considered a risk group, despite reports on biological sex being an important determinant of risk of HIV infection and of subsequent viral pathogenesis, as well as of treatment responses [29].

## Conclusions

The epidemiology of HIV-1-infected women in Israel is changing, showing a shift toward higher prevalence of women from FSU with subtype A HIV-1, infected through heterosexual contact. The proportion of women with any TDRM exceeded 10%, a level which, according to WHO, requires resistance testing, especially as the increase in NNRTI rates (13.3% in 2016–2018) seems to be ongoing. Moreover, when also considering the RT A62 and E138 polymorphic resistance-related sites, as suggested elsewhere [7, 30], the overall prevalence of women with drug-resistance mutations increased to 18.4%, an alarming rate of resistance mutations. These results support the national policy of universal resistance testing soon after diagnosis and call for implementation of appropriate measures, including testing all at-risk pregnant women for HIV-1.

## Supplementary information

**Additional file 1: Table S1.** Predictors of TDRM: univariate and multivariate models.

## Data Availability

The dataset analysed during the current study is available from the corresponding author on reasonable request.

## Abbreviations

TDRM: Transmitted drug resistance mutations
FSU): Former Soviet Union
SSA): Sub-Saharan Africa
PI): Protease inhibitors
NRTI): Nucleotide reverse transcriptase inhibitors
NNRTI): Non-nucleoside reverse transcriptase inhibitors
PR): Protease
RT): Reverse transcriptase
IDUs): Injecting drug users
IQR): Interquartile range
VL): Viral load
