# Supplementary material for: HIV-1 infection among women in Israel, 2010–2018
Source: BMC Infect Dis. 2020 Sep 7;20:660. doi: 10.1186/s12879-020-05389-6 (PMC7487961; doi:10.1186/s12879-020-05389-6)
Supplement: Supplementary file 1 — Additional file 1: Table S1. Predictors of TDRM: univariate and multivariate models. [file 12879_2020_5389_MOESM1_ESM.docx]

|  | **Total TDRM** |  | **NRTI TDRM** |  | **NNRTI TDRM** |  |  |  | **PI TDRM** |  |
| --- | --- | --- | --- | --- | --- | --- | --- | --- | --- | --- |
|  | **Univariate** |  | **Univariate** |  | **Univariate** |  | **Multivariate** |  | **Univariate** |  |
| **Variable** | **OR (95% CI)** | ***p*** | **OR (95% CI)** | ***p*** | **OR (95% CI)** | ***p*** | **OR (95% CI)** | ***p*** | **OR (95% CI)** | ***p*** |
| **Age at diagnosis** | 1.03 (0.99-1.06) | **0.06** | 1.08(1.03-1.14) | **0.002** | 1.03(0.99-1.06) | **0.1** | 1.02(0.99-1.06) | 0.203 | 0.92(0.11-8.01) | 0.939 |
| **Baseline values** |  |  |  |  |  |  |  |  |  |  |
| HIV-RNA load log | 1.04 (0.65-1.67) | 0.86 | 1.09 (0.48-2.49) | 0.838 | 1.08 (0.62-1.87) | 0.79 |  |  | 0.96 (0.30-3.08) | 0.946 |
| Year of diagnosis (2010-2018) | 1.10 (0.97-1.25) | 0.14 | 0.98 (0.78-1.23) | 0.879 | 1.23 (1.05-1.45) | **0.01** | 1.22(1.03-1.43) | **0.020** | 0.87 (0.64-1.19) | 0.38 |
| **Place of birth** |  |  |  |  |  |  |  |  |  |  |
| Sub-Saharan Africa | Reference |  | *** |  | *** |  |  |  | *** |  |
| FSU | 0.60(0.26-1.37) | 0.23 | *** |  | *** |  |  |  | *** |  |
| Israel | 1.31 (0.50-3.44) | 0.58 | *** |  | *** |  |  |  | *** |  |
| Other | 0.30(0.07-1.36) | 0.12 | *** |  | *** |  |  |  | *** |  |
| **HIV-1 subtype** |  |  |  |  |  |  |  |  |  |  |
| A | Reference |  | *** |  | *** |  |  |  | Reference |  |
| C | 1.48(0.67-3.30) | 0.33 | *** |  | *** |  |  |  | 3.77 (0.42-34.14) | 0.238 |
| Non A, C | 1.28(0.47-3.48) | 0.62 | *** |  | *** |  |  |  | 1.99(0.12-32.240) | 0.63 |

Table S1: Predictors of TDRM: univariate and multivariate models

TDRM, transmitted drug resistance mutations; NRTI, nucleoside reverse transcriptase inhibitors; NNRTI, non-nucleoside transcriptase inhibitors; PI, protease inhibitors. p<0.1 was chosen as the cut-off for selecting the potential predictors into the multivariate analyses. In multivariate analysis of NNRTI, the predictor year of diagnosis remained significant after adjusting for the age at diagnosis in years at 0.05 level of significance. *** Regression analysis for place of birth (sub-Saharan Africa, FSU, Israel and other) and for HIV subtype (A, C or non A/C) is not displayed for the all TDRM subgroups and HIV subtype for NRTI and NNRTI subgroups due to uncertainty in model validity (above 95% of women had no NRTI, NNRTI and PI mutations)
